# New Insight into an Old Concept: Role of Immature Erythroid Cells in Immune Pathogenesis of Neonatal Infection

**DOI:** 10.3389/fimmu.2014.00376

**Published:** 2014-08-12

**Authors:** Shokrollah Elahi

**Affiliations:** ^1^Department of Dentistry, Faculty of Medicine and Dentistry, University of Alberta, Edmonton, AB, Canada

**Keywords:** erythroid cells, CD71 cells, innate immunity, neonates, digestive health, microbiota

## Abstract

Newborns are exceedingly susceptible to infection. However, very little is known about what governs the immunological differences seen in early life that result in extreme vulnerability to infection, nor how this changes during infancy. Herein, I provide evidence that the reduced ability to mount a protective immune response to pathogens is not due to an inherent immaturity of neonatal immune cells but instead the functions of these immune cells are actively suppressed by CD71^+^ erythroid cells. Furthermore, the role of CD71^+^ erythroid cells in host defense against infection is examined. CD71^+^ erythroid cells are enriched in newborns and have distinctive immunosuppressive properties that leave them vulnerable to infection. Moreover, immature erythroid cells possess exclusive immunomodulatory properties and may play a role in immune ontogeny. In addition to these distinct features, CD71^+^ erythroid cells impact digestive health by preventing excessive inflammation following the sudden transition from a sterile *in utero* setting to excessive colonization with commensals in the external environment. Ongoing research in identifying the beneficial and/or detrimental effects of immature erythrocytes on immune responses may serve to enhance protective newborn immune responses to infection and enable better vaccination strategies for the young to be designed.

## Introduction

Neonates suffer more severely and die more often than adults from a wide range of infections (1). According to a WHO estimate, almost 7 million children die each year before reaching their fifth birthday (2, 3). Strikingly, two thirds of these deaths are due to infectious diseases (3, 4). While infant mortality is approximately 5 and 7 per 1,000 live births in Canada and the U.S., respectively, the rate is often >30 times higher in developing countries [e.g., 280, 160, 150 per 1,000 in Sierra Leone, Angola, and Congo, respectively (1, 2)]. Even in developed countries, infections in young infants incur an enormous burden. Approximately one infectious disease hospitalization for every 14 infants in the U.S. alone results in an annual cost of approximately $700 million (5). Infectious disease in newborn and infants is a well recognized global issue and the United Nations has outlined a serious of eight millennium development goals to reduce infection-related mortality by two thirds by 2015 (6). However, the mechanisms underlying the susceptibility of neonates to infection and the molecular basis for the transition of immunologic function from fetal to postnatal life have been remained a mystery. The fetus is antigenically different from its mother, and is thus analogous to a semi-allogeneic transplant, with the risk of immunologic rejection. As such, the immune response during pregnancy appears to have evolved to prevent potentially damaging inflammation that may result in spontaneous abortion or preterm delivery (7).

As beneficial as the tolerogenic state might be *in utero*, growing evidence suggests that it predisposes the newborn to severe infections, especially those due to intracellular pathogens, and as well, impairs immune responses to vaccinations in postnatal life (8). Thus, the observed difference in neonatal innate and adaptive immune responses from those elicited later in life and the vulnerability to infection may stem from this fetal tolerogenic state. Moreover, the ability of vaccines to induce protection is increased when immunization starts later possibly due to less interference with maternal immunity (9, 10). However, it has been unclear whether the reduced ability to mount pathogen-specific T and B cell responses is due to an inherent immaturity of effector cells and antigen-presenting cells (APC), or because the functions of these cells are actively inhibited by suppressor cells that are induced during gestation or post-parturition. These discordant observations elucidate the need for a more clarification as to why the immune response to pathogens and vaccines is compromised in the newborn. More recent studies with the discovery of immature erythroid suppressor cells have shown that infection susceptibility in the newborn is not due to an immune-cell-intrinsic defect but instead it is associated with the presence of active immunosuppression mediated by CD71^+^ erythroid cells (11). This finding fundamentally changed the notion of infection susceptibility in newborns by suggesting it is caused by an active immune suppression during this developmental period as opposed to an immune-cell-intrinsic defect. However, CD71^+^ erythroid cell-mediated susceptibility to infection is counterbalanced by CD71^+^ erythroid cell protection against aberrant immune cell activation in the intestine, where colonization with commensal microorganisms occurs swiftly after parturition (11). Therefore, better understanding the role of CD71^+^ erythroid cells in imprinting the immune cells that affect the generation and maintenance of protective immunity to infection will assist in the design of better interventions to improve the quality of life in this most vulnerable population.

The purpose of this review is to examine existing evidence regarding the immunological properties of immature erythroid cells and to highlight their role in immune pathogenesis of prenatal infection. I will discuss distinct aspects of immunosuppression associated with erythropoiesis in particular CD71^+^ erythroid cells mainly on innate immunity. Next I will explore the cross talk between CD71^+^ erythroid cells and gut microbiota and how this interaction could affect intestinal health. Lastly, I will discuss future directions in the field and elaborate on further studies for novel therapeutic possibilities aimed at dissociating the beneficial and harmful effects of CD71^+^ erythroid cells in augmenting host defense against infections in the newborn.

## Nucleated Erythrocytes in Vertebrates

The main function of vertebrate erythrocytes is considered to be oxygen-transport, however, other functions such as interactions with immune cells have also been assigned to these cells (12). Nucleated erythrocytes in vertebrate mammals are defined as immature erythroid cells and are seen mainly in newborns, but interestingly, these cells are abundant throughout the life cycle of non-mammalian vertebrates (12).

Intriguingly, nucleated erythrocytes from non-mammalian vertebrates such as fish and birds express and regulate specific pattern recognition receptors (PRR), including members of toll-like receptors (TLRs) and peptidoglycan recognition protein (PGRP) receptor families, and are also capable of specific pathogen-associated molecular pattern (PAMP) recognition that is instrumental to the innate immune response (13–15). Furthermore, it has been shown that erythrocytes constitutively express transcripts for different TLRs and respond to TLR ligands by up-regulating type I IFN, IL-8, CCL-4, as well as nitrite production in non-mammalian vertebrates (13, 15). The production of type I IFN is a well-defined immune response that bridges the innate and adaptive immune responses (16). Thus, an essential role in pathogen recognition must be given to non-mammalian nucleated erythrocytes. However, the potential contribution of mammalian nucleated erythrocytes in non-respiratory physiological processes such as immune-regulation is not very well appreciated. Therefore, studies aimed at further characterization of immunological role of nucleated erythrocytes in mammalian vertebrates are crucial.

## Immunosuppression and Immunomodulation Mediated by Nucleated Erythrocytes

In 1979, the concept of immunosuppression mediated by splenic nucleated erythrocytes was revealed for the first time (17, 18). These studies showed that nucleated erythrocytes were able to suppress primary and secondary antibody-mediated responses *in vivo* (17, 19). Later on, these nucleated erythrocytes were described as erythroid immunosuppressor cells (ESC), capable of inhibiting B cell proliferation and humoral immune responses both in mice and human beings (20). Interestingly, these studies noted that erythropoietic disturbances lead to the appearance of immature red blood cells and subsequently resulted in the suppression of B cell proliferation in the peripheral blood and lymph nodes (20). The mechanism by which ESC-mediate immunosuppression was reported to be associated with soluble factors. For example, it was reported that erythroid precursors produce a species-non-specific type of soluble factor (1–10 kD) that suppresses both IgM and IgG secretion and proliferation of human B cells (21). In line with these observations, Seledtsova et al. found that ESC not only exert suppression of LPS-driven B cell proliferation but also inhibit proliferative cytotoxic T cell responses (22). Although the underlying mechanism(s) of suppression was not clearly determined, their data indicated that a soluble heat stable molecule (80°C for 20 min) with low molecular weight was capable of effectively reducing the allogeneic-driven proliferation of peripheral blood mononuclear cells (PBMC) isolated from allergic patients (22). Subsequent studies by Seledtsova et al. indicated that the immunosuppressive activity of ESC might be partially mediated through TGF-β (23) and direct cell–cell interactions (24).

A second important piece of immature erythroid cell functionality relates to their ability to produce cytokines or other immune-modulatory molecules. Although, hematopoiesis is regulated through a complex network of paracrine and autocrine mechanisms involving cytokines, growth factors and their receptors (25), erythroid-sourced cytokines in the microenvironment can have potential immunomodulatory effects on other non-hematopoietic cells such as immune cell lineages.

Patterns of cytokine production and the cytokine milieu can promote polarization of naive CD4 T cells into distinct Th1, Th2, or Th17 subtypes (26). Presence of regulatory cytokines such as IL-10 and TGF-β could also induce and expand regulatory T cells (Tregs) (26). In fact, it has previously been reported that ESC from newborn mice are capable of expressing a wide array of mRNA cytokines such as IL-1α, IL-1β, IL-4, IL-6, and GM-CSF (27). Similar studies have shown that human bone marrow-derived erythroid nuclear cells produce a wide range of cytokines such as IL-1β, IL-2, IL-4, IL-6, IFN-γ, TNF-α, TGF-β, and IL-10 (25, 28), suggesting that these cells can respond to microenvironmental changes by altering their cytokine production profile. Recently, up-regulation of IL-4 expression in activated CD4^+^ T cells co-cultured with immature erythroid cells was noted in newborn mice (29). Further studies by this group indicated that the immature erythroid cells have the ability to produce IL-6 during activation of CD4 T cells, which contributes to IL-4 up-regulation in CD4 T cells and therefore promotes a bias toward Th2 phenotype effector cells in neonatal mice (29).

More recently, arginine depletion has been documented as a mechanism of suppression by immature erythroid cells (11), similar to the suppressor cells that are associated with persistent infection (30). Interestingly, the suppression by neonatal but not adult immature erythroid cells parallels the markedly higher expression of arginase-2 in these cells compared to immature erythroid cells obtained from adult phlebotomized mice (11).

Although, some discrepancies can be seen between the data from different groups, in my view, such inconsistency in cytokine production capabilities of immature erythroid cells can be explained by multiple factors: (a) The cell source; immature erythroid cells originating from different organs might be an important factor to consider when interpreting the data, for instance human embryonic liver cells and/or bone marrow-derived cells versus cord blood immature erythroid cells. (b) The experimental approach and stimulus; for instance, the type of stimuli, whether testing a single cell population versus whole splenocytes and the duration of stimulation could all impact the outcome. (c) The heterogenicity of immature erythroid cells; lack of defining markers for immature erythroid cells in the previous studies might have impacted their outcome. Future studies aimed at immature erythroid cells require using CD71TER119 and CD71CD235a markers for mice and human beings, respectively.

Despite multiple supportive reports regarding immunosuppressive properties of immature erythroid cells, further comprehensive studies aimed cytokine production capabilities of CD71^+^ erythroid cells originated from different organs are required. In addition, utilizing deep RNA sequencing may provide some clues if there are base-line differences in the transcriptomes from CD71^+^ cells sourced from different organs such as fetal liver, neonatal spleen, and adult spleen.

**Figure 1:**
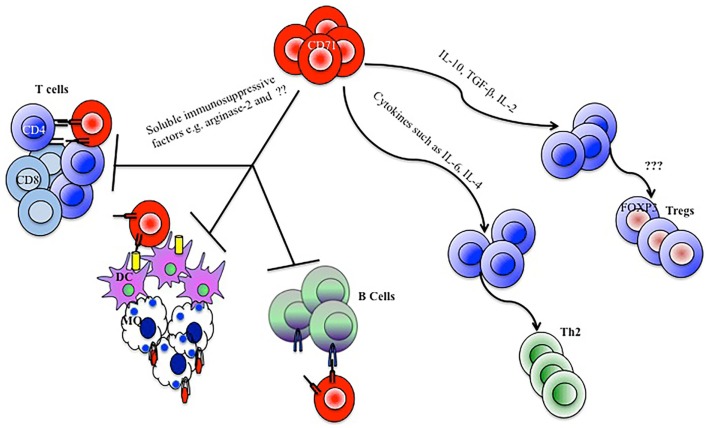
**Model depicting how CD71^+^ erythroid cells mediate immunomodulatory functions**. CD71^+^ erythroid cells by secreting soluble immunosuppressive factors, depletion of arginine, and direct cell–cell contact manners inhibit CD4, CD8, B cell, macrophage (MQ), and dendritic cell (DC) responses. CD71^+^ erythroid cells could also skew a Th2 type immune response or expand regulatory T cells (Tregs), by the production of cytokines and influence on the cytokine milieu.

Taken together, on one hand, nucleated erythrocytes through cytokines, arginase-2, other possible unidentified soluble factors, and cell–cell contact-dependent manners suppress innate and adaptive immune responses (immunosuppressive effects). On the other hand, they might create a balanced mediator microenvironment providing the necessary signals required for natural development of different hematopoietic and immune cell lineages (immunomodulatory effects) (Figure 1).

## Impact of Nucleated Erythroid Cells on Neonatal Innate Immune System

Neonates are thought to be significantly dependent on their innate immune system for protection against invasive pathogens. The innate immune response not only plays an instrumental and non-antigen specific protective role against pathogens in the newborn (7), but also through the interaction with T and B cells, regulates tolerance to self and generates immune responses to vaccines and memory responses to antigens. Although the immunosuppressive activity of murine neonatal splenocytes co-cultured with adult lymphocytes was first reported by Pavia et al. (18), this field has since received limited attention. As described above, immature erythroid cells possess the capability of secreting a wide range of cytokines. Induction of cytokines by immature erythroid cells following innate immune stimulation can influence the cytokine milieu and could contribute to the differentiation and function of immune cells. In agreement, it has been reported that nucleated immature erythroid cells (expressing erythroid marker TER119, LY-76) are capable of secreting cytokines such as IL-6 which alters the Th1/Th2 cytokine balance and contributes to the induction of a Th2 phenotype observed in the neonate (29).

Most recent observations in the field have demonstrated that erythroid precursor cells are abundant in the neonatal spleen. They co-express the transferrin receptor CD71 and the erythroid lineage marker TER119 (11). However, they gradually decay and reach to the adult levels by day 21 post-parturition (11). Similarly, human cord blood contains an equally enriched proportion of erythroid precursor cells co-expressing CD71 and the erythroid marker CD235a (11). Accordingly, as discussed above, these cells have distinctive immunosuppressive properties by diminishing the production of innate immune cytokines by adult cells following adoptive transfer into neonatal mice and also *in vitro*, co-cultured with adult or neonatal cells. Remarkably, their presence after birth inhibits innate immune responses and contributes to the susceptibility of newborns to perinatal pathogens such as *Listeria monocytogenes* and *Escherichia coli* (11). More importantly, the ablation of CD71^+^ erythroid cells in neonates using neutralizing antibody, or the decrease in accumulation of these cells in spleen as postnatal development progresses parallels the loss of suppression and restored resistance to perinatal pathogens (11). It is important to mention the possibility of CD71 expression by other immune cell lineages in the neonate of both mice and human beings. Thus studies to determine the inclusion or exclusion of non-erythroid CD71 + cells using anti-CD71 antibodies are required.

Taken together, these data demonstrated that neonatal infection susceptibility results from the temporal presence of erythroid immunosuppressive CD71^+^ cells. This in fact challenges the notion that the susceptibility of neonates to infection reflects immune-cell-intrinsic defects bust instead highlights the presence of active immune suppression in this developmental period. One of the caveats of the above study is that the intraperitoneal infection route was utilized, since vast majority of infectious agents enter the body via mucosal surfaces notably the lungs and intestines. Thus, it remains to be determined whether these mechanisms also regulate immune responses to pathogens when infected at mucosal sites, and whether these cells compromise innate immune response to viral infections. Furthermore, it is important to investigate whether these cells exhibit other immunomodulatory properties by secreting cytokine, chemokine, and/or other soluble factors. Finally, further investigation aimed at studying the frequency and function of these cells in newborns may explain some of the distinct and unpredicted immune responses seen in human newborns and infants.

## Impacts of Nucleated Erythroid Cells on Adaptive Immune System

Newborns are restrained from exposure to antigens *in utero* to generate adaptive immunity. The adaptive immune system consists of cell mediated and antibody-mediated responses. It is plausible to predict that nucleated erythroid cells may impact the development and function of peripheral helper T cells, cytotoxic T cells, and also B cells to mediate acquired immunity.

Early studies on nucleated erythrocytes demonstrated that these cells are capable of inhibiting B cell proliferation and humoral immune response both in mice and human beings (20). Interestingly, these studies noted that erythropoiesis disturbances lead to the appearance of immature red blood cell precursors and subsequently result in the suppression of B cell proliferation in the peripheral blood and lymph nodes (20). In agreement, Mitasov et al. reported that immature murine erythroid cells suppress both IgM and IgG secretion and the proliferation of B cells (21). In line with these observations, Seledtsova et al. found that nucleated erythroid cells not only exert suppression of LPS-driven B cell proliferation but also proliferative cytotoxic T cell responses (22). More recent studies indicated that CD71^+^ erythroid cells hinder up-regulation of early activation markers (e.g., CD25 and CD69) among T cells following anti-CD3 antibody stimulation *in vitro* (11). These studies underline the sophisticated nature of the immune response in neonates and suggest further required investigation on the effects of immature erythroid cells on adaptive immune response in the newborn.

## Erythroid CD71^+^ Cells and Digestive Health

Under normal circumstances, the fetal gastrointestinal tract is supposed to be sterile, with the initial exposure of the newborn’s mucosa to commensals taking place during passage through birth canal and exposure to outside environment as well as milk. Colonization with a diverse variety of microorganisms is critical for the normal development of the newborn’s commensal microbiota (31). Although the early interactions between the host and the microbiota are considered to set the tone for the mucosal and systemic immune system for the long term, the acquisition of a diverse microbiota following the dramatic transition from a sterile *in utero* setting to excessive colonization with these commensals in the external environment can be tricky and may come at a price. Birth triggers a sudden shift that challenges the newborn’s immune system with a massive influx of foreign antigens including microorganisms, yet a pro-inflammatory response against these new invaders will be counterproductive and detrimental. The mechanism by which the neonate mucosal surfaces, in particular the gastrointestinal tract, adapt to the alarming challenge of microbial colonization has remained a mystery. In this context, more recent studies have revealed that CD71^+^ erythroid cells mediate a swift transition from a sterile intrauterine compartment to the non-sterile external environment, as depletion of CD71^+^ erythroid cells results in excessive inflammation in newborn mice (11). In particular, intestinal CD11b^+^ and CD11c^+^ cells from CD71^+^ cell-depleted neonatal mice produce significantly more TNF-a and up-regulate expression of the co-stimulatory molecules CD40, CD80, and CD86 compared with controls. By contrast, depletion of CD71^+^ erythroid cells did not alter the activation status of immune cells in the spleen and lung, which remain sterile or transiently become colonized with fewer commensals. Interestingly, CD71^+^ erythroid cell depletion did not induce significant alteration in intestinal immune cell activation in germ-free and antibiotic-treated neonatal mice (11), demonstrating cross talk between CD71^+^ erythroid cells and immune cell lineages in the gut.

Therefore, neonatal CD71^+^ erythroid cells contribute to the maintenance of this immunoregulatory environment by limiting the surge of mucosal pro-inflammatory signals and allowing swift adaptation to its newfound allies (11). An immune response against colonization of commensals in the neonate could interfere with establishing a symbiotic equilibrium and subsequently could compromise the fundamental role of microbiota in shaping the immune system of the infant (32). Taken together, the higher susceptibility of newborns to perinatal pathogens seems to be a by-product of the greater benefits of active immune suppression during this crucial developmental period, when tolerance to colonization with commensals is more constantly propitious.

In addition to CD71^+^ erythroid cells, the transfer of antibodies, live microbes and immune cells as well as cytokines via colostrum and breast milk, plays an important role in shaping the composition of the gastrointestinal tract microbiota (31). These factors synergize to shape the breast-fed infant micobiota and the immune response of the host to these microbes. For instance, IgA limits immune activation and microbial attachment by binding microbial antigens, and the presence of metabolites including oligosaccharides in mother’s milk promotes the expansion of defined constituents of the microbiota such as *Bifidobacterium* (33, 34).

**Figure 2:**
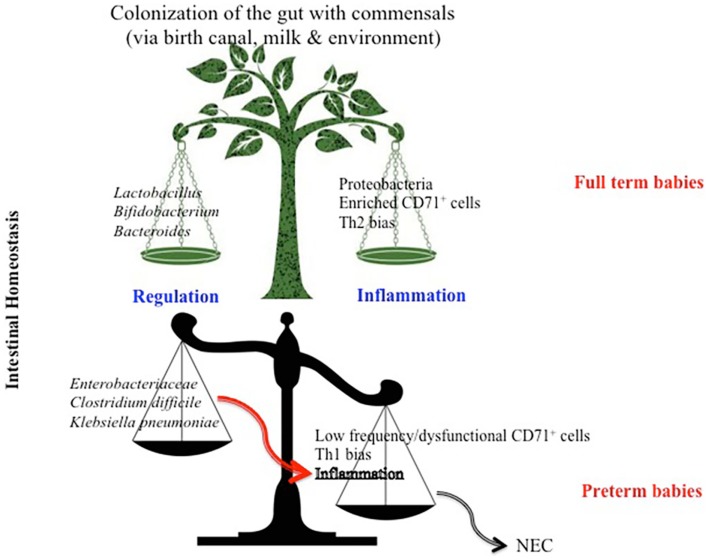
**Model depicting differential factors influencing intestinal homeostasis in full term versus preterm newborns**. High proportion of CD71^+^ erythroid cells in full term infants skews their immune response toward a Th2 phenotype which influences the bacterial composition to a diverse commensal colonization and subsequently a regulated intestinal immune response. Whereas, lower frequency of CD71^+^ erythroid cells in preterm infants results in a Th1 bias immune response, which favors colonization of the gut with more pathogenic bacteria, subsequently predisposes the preterm to necrotizing entrocolitis (NEC).

Although *Bifidobacterium* is a dominant bacterial genus in the neonatal gut microbiota (32), gestational age is also an instrumental factor that strongly influences the subsequent establishment of the infant’s intestinal microbiota. Comparison of the fecal microbiota of full term and preterm infants has revealed significant niche differences. In full term infants, the diversity of the fecal microbiota is greater and more common genera such as *Bifidobacterium*, *Lactobacillus*, and *Streptococcus* are present (35) whereas Enterobacteriaceae and other potentially pathogenic bacteria such as *Clostridium difficile* or *Klebsiella pneumoniae* are found in greater numbers in preterm infants (36). Different *Lactobacillus* species activate dendritic cells, inducing them to produce different arrays of inflammatory cytokines, thus playing a major role in the modulation of the Th1, Th2, and Th3 balance (37, 38). Therefore, *Lactobacillus* species can augment host intestinal defenses by promoting anti-inflammatory signaling, blocking inflammatory signaling, and improving gut barrier function (39). In contrast, dominance of Enterobacteriaceae and *C. difficile* in preterm infants diminishes intestinal host defense mechanisms by a Th1 inflammatory response (40) and may result in pathological alterations such as Necrotizing enterocolitis (NEC) (Figure 2).

In preterm babies, NEC is the most devastating gastrointestinal emergency with high morbidity and mortality (41). Unfortunately, despite extensive clinical and basic research, the etiology and the exact mechanism(s) underlying the inflammation and injury leading to NEC are poorly understood. However, the lower bacterial diversity and presence of pathogenic bacteria may contribute to increased apoptotic signaling, enhanced inflammatory response, and a reduced gut barrier function which predisposes the preterm to NEC (42). In addition, the expression of pro-inflammatory cytokines such as TNF-α has been associated with NEC pathogenesis (43).

**Figure 3:**
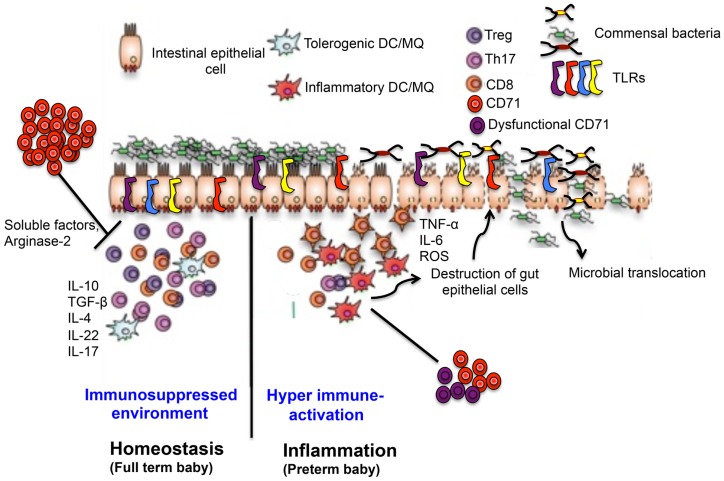
**Proposed mechanisms of CD71^+^ erythroid cells-induced gut immune-regulation in full term versus preterm newborns**. In full terms, enriched CD71^+^ erythroid cells generate a suppressed immune environment by regulatory and Th2 type cytokine in the intestine, which down-regulates TLR expression, maintains symbiosis and intestinal integrity. In contrast, lower and/or dysfunctional CD71^+^ erythroid cells in preterm disrupts normal immune homeostasis in the gut leading to a switch from a suppressed environment to a pro-inflammatory state, up-regulates TLR expression, dysbiosis, and pathological alterations associated with NEC.

Of note, the cytokine profile toward Th1, Th2, or Th17 predominance has been shown to contribute to chronic inflammatory bowel disease in human beings and animal models and likely influences TLRs expression in the intestinal mucosa (44). Although, TLR signaling is tightly regulated and coordinates homeostatic responses to commensal bacteria (45, 46), Th2 cytokines, and predominantly IL-4, and it appears to dampen TLR expression and function in human intestinal epithelial cells (IECs) (45). Therefore, it is possible to predict that the selective accumulation of CD71^+^ cells may explain the apparent differences observed in the type of immune responses (Th2) generated in neonates (29). Consequently, the Th2 phenotype down-regulates expression of TLRs in order to quench the excessive inflammation induced by sudden colonization with commensal bacteria after parturition (Figure 3). It has been reported that the premature intestinal environment is predisposed to exaggerated inflammatory responses, possibly leading to NEC (47, 48). Because host-mediated inflammation alone is sufficient to perturb the composition of the intestinal microbiota, it eliminates a subset of bacteria while supporting the growth of others (40). With this concept, it is plausible to hypothesize that the hyperinflammation leading to destruction of the intestine seen in NEC in premature infants might take place because the immune system of the infant overreacts to the commensal colonization as the immunosuppressive CD71^+^ erythroid cells has yet to be developed. Specifically, nucleated erythroid cells can produce cytokines that contribute to the Th1/Th2 balance, an important one being IL-6 (29). As IL-6 has pro-resolution properties, including inhibition of neutrophil migration (49), this polarization may serve to reduce the risk of an excessive pro-inflammatory/Tn1 response during the initial colonization of the gut with microbiota. Further studies are required to determine whether CD71-depletion in full term newborn results is dysbiosis or any histopathological alterations such as increased intestinal permeability and subsequent bacterial translocation.

## Conclusion Remarks

Susceptibility of newborn to infection stems from the accumulation of immature erythroid cells in this stage of life. These findings highlight the pivotal role of CD71^+^ erythroid suppressor cells in compromising the innate immune response and immune ontogeny, which makes them the primary target cells for enhancing the immune responses against infection. Further investigations aimed at dissociating the beneficial/detrimental role of CD71^+^ erythroid cells for possible therapeutic approaches are required. Ongoing studies characterizing the effects of CD71^+^ erythroid cells on neonatal innate and adaptive immunity and assessing potentially beneficial effects of these cells on prevention of NEC will inform efforts to modify or enhance immune response in this most vulnerable population. Using animal models with defined genetic deletions and high-throughput sequencing techniques could shed some light on the cross talk between CD71^+^ erythroid cells with other immune cells and their influence on TLR signaling in the gut.

## Conflict of Interest Statement

The author declares that the research was conducted in the absence of any commercial or financial relationships that could be construed as a potential conflict of interest.
